# Supplementary figures and images for: Transcriptome and Proteome Co-Profiling Offers an Understanding of Pre-Harvest Sprouting (PHS) Molecular Mechanisms in Wheat (Triticum aestivum)
Source: Plants (Basel). 2022 Oct 22;11(21):2807. doi: 10.3390/plants11212807 (PMC9657071; doi:10.3390/plants11212807)

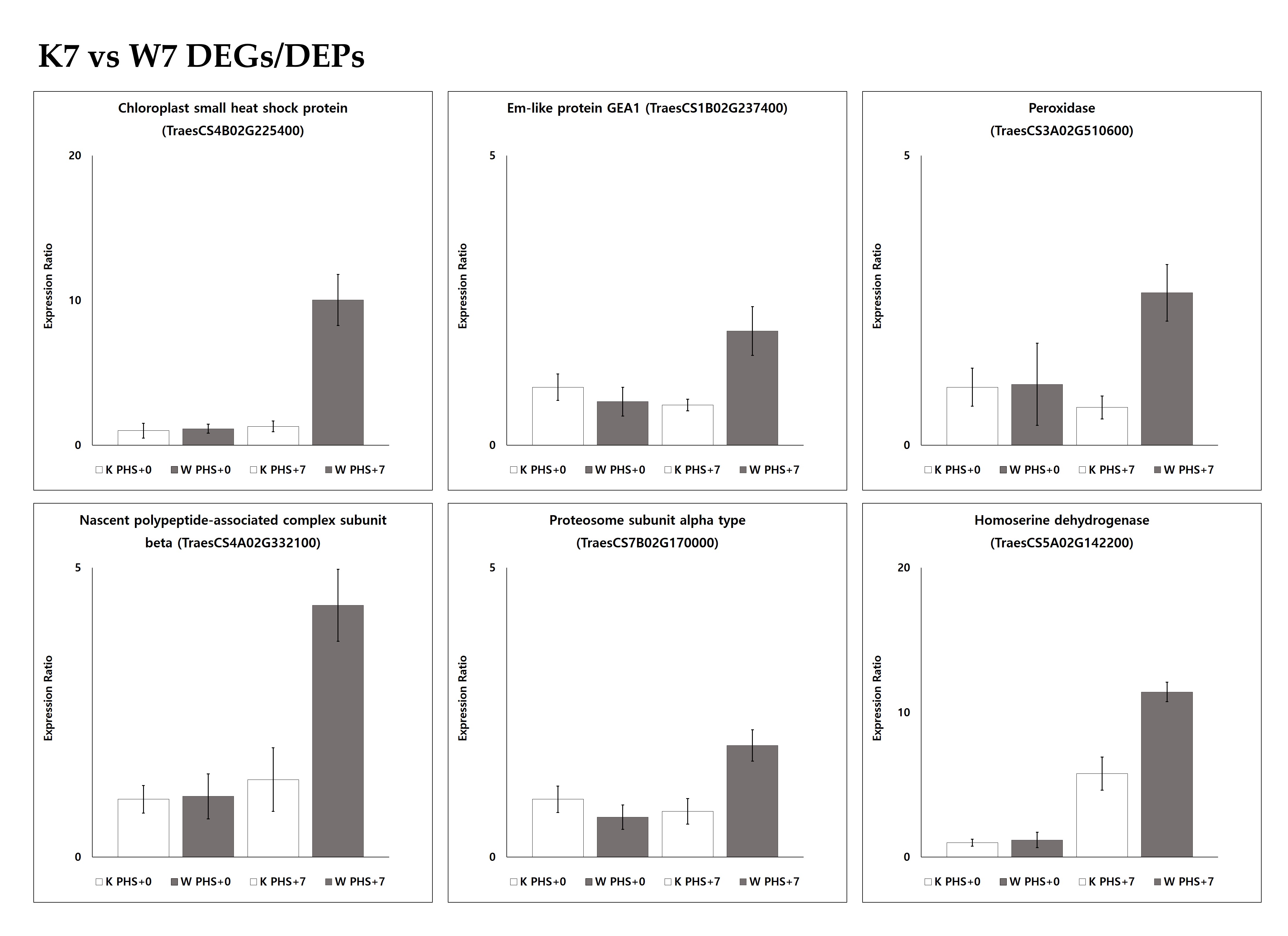

Supplement: Supplementary file 1 [file plants-11-02807-s001.zip › supplementaryinformation/Supplementary Figure S1.jpg]

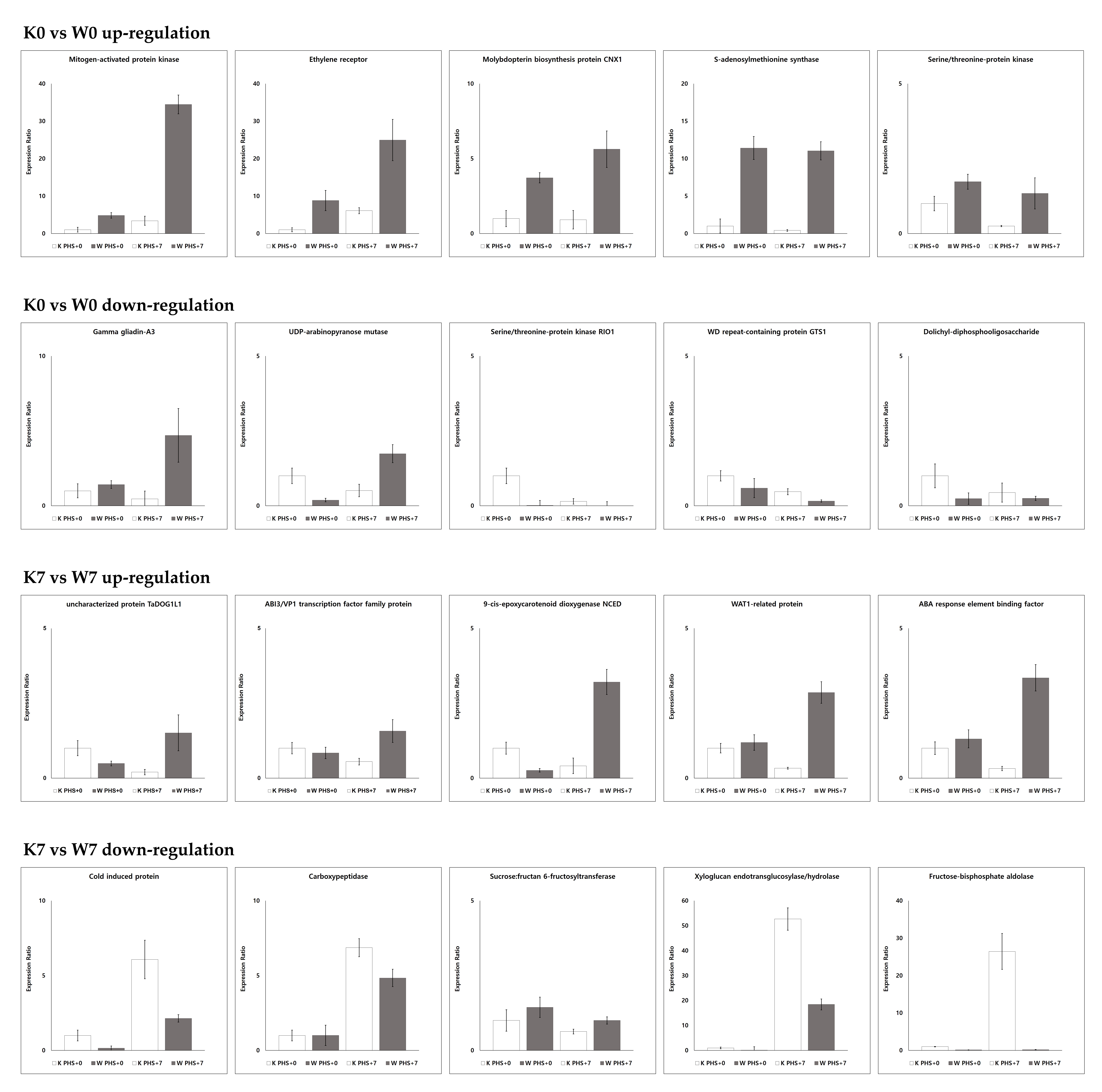

Supplement: Supplementary file 1 [file plants-11-02807-s001.zip › supplementaryinformation/Supplementary Figure S2.jpg]

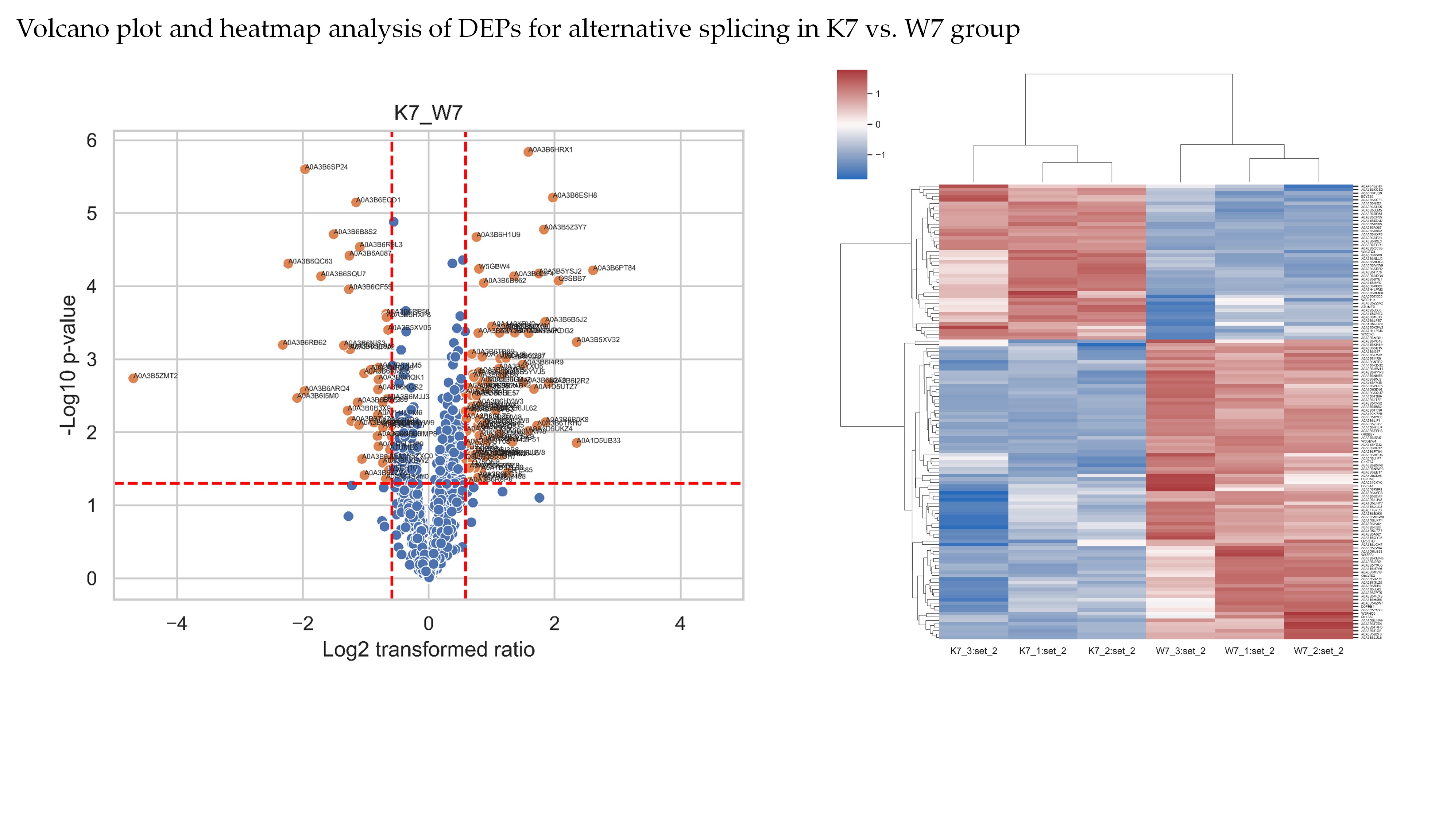

Supplement: Supplementary file 1 [file plants-11-02807-s001.zip › supplementaryinformation/Supplementary Figure S3.jpg]

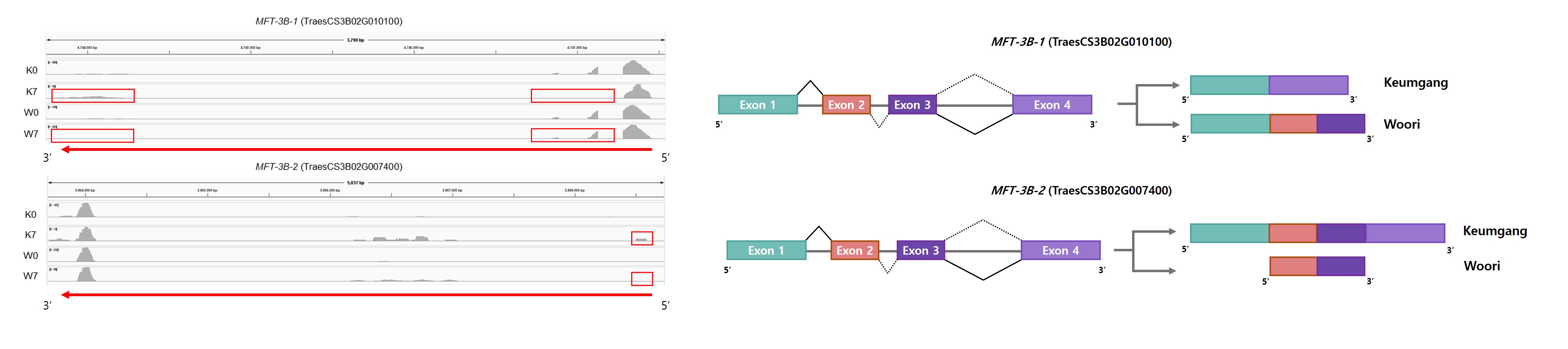

Supplement: Supplementary file 1 [file plants-11-02807-s001.zip › supplementaryinformation/Supplementary Figure S4.jpg]

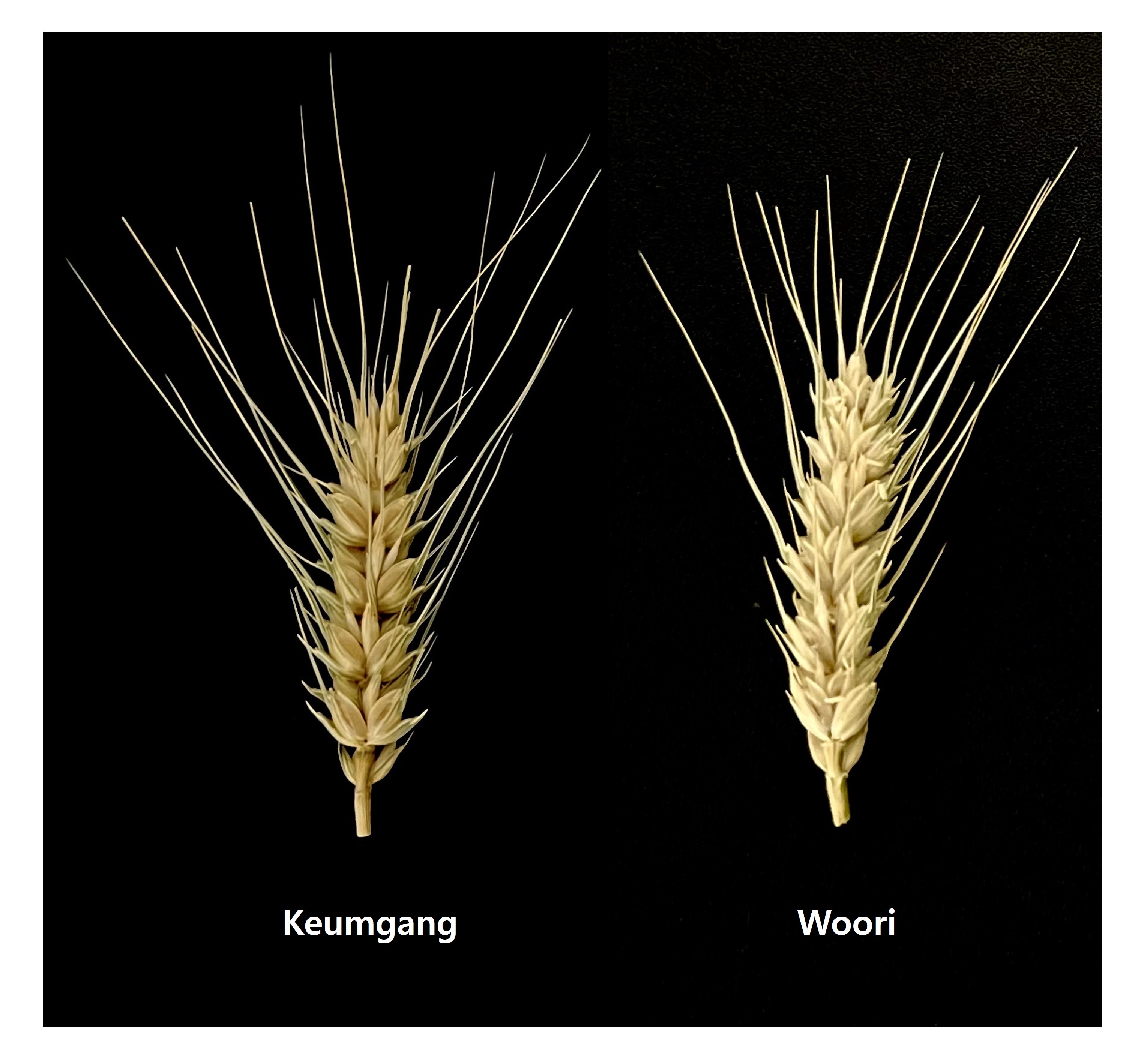

Supplement: Supplementary file 1 [file plants-11-02807-s001.zip › supplementaryinformation/Supplementary Figure S5.jpg]
